# 1210. A Lean Six Sigma Approach to Improving Accuracy of Manual Hand Hygiene Observations

**DOI:** 10.1093/ofid/ofac492.1043

**Published:** 2022-12-15

**Authors:** Marci Drees, Kathleen Bonis, Krystal Coles, John Emberger, Lauri Littleton, Mark Mirage, Austin Mount-Campbell, Carol Briody

**Affiliations:** ChristianaCare, Newark, Delaware; ChristianaCare, Newark, Delaware; ChristianaCare, Newark, Delaware; ChristianaCare, Newark, Delaware; ChristianaCare, Newark, Delaware; ChristianaCare, Newark, Delaware; ChristianaCare, Newark, Delaware; ChristianaCare, Newark, Delaware

## Abstract

**Background:**

Hand hygiene (HH) is widely regarded as the most important factor in preventing transmission of infections. Since 2012 our health system has utilized unit-based direct observation to measure HH compliance. Although direct observation is widely used and considered gold standard, the discrepancy between unit-based HH compliance (UB-HH) and Infection Prevention validation HH audits (IP-HH) was increasing over time. To understand the drift in HH compliance, we began a Lean Six Sigma (LSS) Green Belt project to improve UB-HH observation accuracy.

**Methods:**

The IP LSS Green Belt team included nursing, respiratory care, and human factors, and analyzed factors leading to inaccurate UB-HH using LSS tools including the Voice of the Customer, process mapping, fishbone diagrams, and failure modes and effects analysis. We updated HH observer web-based education; implemented a new process to ensure observer training; and eliminated unit report card penalties for poor UB-HH. We implemented a new, more accessible observation tool, which provides a dashboard for real-time access to HH results by all staff. IPs began weekly validation HH audits.

**Results:**

Baseline data revealed a 34% discrepancy between UB-HH and IP-HH compliance (95% vs 61%) over 4 different monthly validation events; only 27% of observers had completed web-based training. Goal conflicts were discovered: units were penalized for poor HH, yet the observations were unit level self-report. These results prompted design changes to the online tool and the process flow of UB-HH observation; units implemented the new program sequentially between 9/20 and 12/20. These changes resulted in 99% of observers being web-trained; however, between 10/21-3/22, UB-HH compliance averaged 98% (n=19,940), while IP-HH compliance averaged 53% (n=579) (difference, 45%).

**Conclusion:**

Using multidisciplinary process improvement, we enhanced our manual HH observation processes; however, no improvement in HH accuracy was observed. Unit-based staff, who lack dedicated time for HH observation, are biased to document HH compliance over non-compliance, even with recent re-training in non-biased HH observation processes and elimination of penalties. To improve HH accuracy, we recommend either dedicated neutral HH observers or automated systems.

**Disclosures:**

**All Authors**: No reported disclosures.

